# Supplementary material for: A set of multi-entry identification keys to African frugivorous flies (Diptera, Tephritidae)
Source: Zookeys. 2014 Jul 24;(428):97–108. doi: 10.3897/zookeys.428.7366 (PMC4143993; doi:10.3897/zookeys.428.7366)
Supplement: Supplementary material 4 — Key to Capparimyia [file zookeys-428-097-s004.zip › SF4_ZooKeys_key to Capparimyia/lucid_player/help/help/whgdata/whlstt0.htm]

Content


Help for the Lucid3 Player  
 What does the Lucid3 Player do?  
 Registration and Activation  
 The Applet Player and Application Player  
 System Requirements  
 Using the Lucid3 Player  
 The Lucid Player�s Menu and Button Bar  
 About the Lucid Family of Products  
 About the Lucid3 Builder
